# Is it time to rethink UK restrictions on blood donation?

**DOI:** 10.1016/j.eclinm.2019.10.014

**Published:** 2019-10-29

The WHO Melbourne declaration set forth the target that by 2020 all countries obtain 100% of blood supply from unpaid voluntary donors. To ensure that the supply of blood is safe, health services impose a wide range of restrictions on the individuals who can donate. With the recent announcement by the Irish Blood Transfusion Service to relax restrictions on formerly UK-based blood donors, is it time to rethink the restriction on individuals who are still unable to donate?

When donating blood in the UK, the health screening includes a question on whether the individual has had a blood or blood product donation. This question exists, among other reasons, because of safety concerns surrounding the bovine spongiform encephalopathy/variant Creutzfeldt-Jakob disease (BSE/vCJD) outbreak of the early to mid 1990s. BSE, colloquially referred to as mad cow disease, is a neurodegenerative disorder affecting bovine livestock caused by the recycled use of animal remains in livestock feed. The causative agents are misfolded prion proteins, which are transferred in the consumption of infected tissues. The widespread transmission of the disease in cattle, and subsequent human consumption of infected beef resulted in the contraction of a variant of the human disorder, Creutzfeldt-Jakob disease (vCJD). This fatal disorder, in its spontaneous form, was typically observed in people older than 60 years, at a very small incidence. vCJD affects various aspects of neurological function, including memory, location, and sight, always resulting in death. The variant form of the disorder has, to date, resulted in approximately 178 deaths. To curb the disease, the UK Government culled and destroyed more than 4 million cows. Internationally, UK bovine products were banned from export to most countries for at least a decade.

Alongside the immediate problem of animal to human transmission were the potential long-term concerns of human to human transmission. Iatrogenic transmission occurring from the reuse of medical equipment used on individuals with CJD was a concern and was curtailed by more rigorous sterilisations and usage guidelines. Although vCJD cases resulting from organ transplantation had arisen, zero cases from blood transfusion had been reported in humans. Concerns over the long-term incubation period of the disease led to estimates of a potentially huge latent pool of future sufferers. By 2000, a number of animal studies had shown that the prion proteins responsible for CJD, if found in blood used for transfusion, were able to directly transfer the disorder between individuals. The combination of these pieces of information began to shift members of public health advisory groups towards increased stringent regulations. Subsequently, Switzerland issued a ban on blood donors who had spent more than 6 months in Britain during the original outbreak. This ban was also implemented in New Zealand and was followed shortly thereafter by bans in a a large number of countries. By 2004, the number of cases of vCJD caused by consumption of infected beef decreased significantly to controlled levels. However, 2004 also saw the emergence of a small number of individuals developing vCJD after having received blood transfusions from as-yet-undiagnosed individuals with vCJD. This resulted in a second national alert that, combined with research predicting large numbers of individuals who had potentially been infected after blood transfusion, prompted the then UK and Irish Governments to enforce guidelines that are still in place today. These guidelines included use of leucodepletion to remove white blood cells from blood products and, most importantly, the restriction of blood donation by individuals who had received transfusions from the 1980s onwards. These guidelines were further influenced by the potential for outbreak of other bloodborne diseases such as HIV and hepatitis B virus.

To date, four individuals who, having received blood transfusions from people who subsequently died of vCJD, died themselves from vCJD. Although any deaths are cause for concern, the relatively low incidence of transfusion-related death indicates that the actual risk to the public is low. Indeed, news announced last month by the Irish Blood Transfusion service (IBTS) indicated their intention to reverse their ban on UK blood donations. Introduced in 2004, the ban prevented individuals who had lived in the UK between 1980 and 1996 from donating blood. Director of the IBTS, Stephen Field, pointed out the massive discrepancy between the predicted number of transfusion-related cases and actual cases. Alongside this, he pointed out that the four known cases had occurred before the implementation of the leucodepletion preventative measure, further confirming the low current risk of transmission. This change in policy is a significant step towards the reassessment of the guidelines in other countries. The immediate hopes of the referral will be to reclaim some of the 10 000 lost donors excluded over the period of the ban in Ireland alone. This situation is similar to the changes in regulation of donation from men who have sex with men (MSM) as a concern over potential sexually transmitted disease (STI) transmissions. In most countries, MSM can now donate, although they must undergo strict lifestyle questioning and often deferral periods before blood product use. The major difference between restrictions on MSM and the vCJD restrictions being the presence of reliable blood tests for many of the concerning viral pathogens which cause STIs not currently available for prions.

For many, vCJD is an emotional and controversial issue. At the time of the initial UK BSE outbreak, government officials were, at best, slow and, at worst, negligent in their actions to tackle the effects on humans. Famously, the agriculture minister, John Gummer sought to quell concern by publicly encouraging his infant daughter to eat a beef burger, only for the government to recognise the danger posed and initiate the mass cull of cows 6 years later. By this time, millions had been further exposed, justifiably causing an evaporation of reassurance in the government and, by association, the scientific community to protect the public. Nearly 30 years on there is still some push back in relaxing safeguards associated with vCJD. In addition, the current blood contamination inquiry looking at the potential infection of 30 000 individuals during the 1970s and 1980s is creating further controversy around the safety of blood products in the NHS. These issues suggest that, moving forward, the issue must be addressed with cautious revaluation.

Donating blood is a hugely rewarding experience and, in an ideal situation, should be available to as much of the population as possible. Combined with the WHO declaration on blood donation, many countries are in the midst of a difficult situation to reach voluntary donor targets. It is a sad irony that the individuals who might have benefitted the most from blood donation, who are often the most eager to reciprocate, are still unable in the UK. Further research into treatments for vCJD are essential. The lack of a reliable blood test is crippling, and second only to the lack of a treatment in the importance of issues surrounding this disease. Journals such as ours focus on encouraging and nurturing the dissemination of information with the hope that this knowledge gap can be bridged. The balance of public safety with the loss of benefits to a society can be a difficult balancing act. However, it is essential that authorities constantly seek to ensure they are using the most up to date data available and are quick to act as the risk:benefit balance of a disease evolves.

*EClinicalMedicine*

